# De Novo Biopsy-Proven Glomerular Disease Following COVID-19 Vaccination

**DOI:** 10.3390/jcm13154494

**Published:** 2024-07-31

**Authors:** Cheng-Hsu Chen, Yu-Wei Chiu, Bo-Ding Chen, Ming-Ju Wu, Shang-Feng Tsai

**Affiliations:** 1 Division of Nephrology, Department of Internal Medicine, Taichung Veterans General Hospital, 160, Sec. 3, Taiwan Boulevard, Taichung 407, Taiwancyw9801319@gmail.com (Y.-W.C.);; 2 Department of Life Science, Tunghai University, Taichung 407, Taiwan; 3 Department of Post-Baccalaureate Medicine, College of Medicine, National Chung Hsing University, Taichung 402, Taiwan; 4 PhD Program, Tissue Engineering and Regenerative Medicine, College of Medicine, National Chung Hsing University, Taichung 402, Taiwan; 5 Shinfu Clinic, Taichung 411, Taiwan; 6 Guomao Clinic, Taichung 436, Taiwan; 7 Department of Medicine, National Yang-Ming University, Taipei 112, Taiwan

**Keywords:** COVID-19 vaccine-associated glomerular disease (CVAGD), glomerular disease (GD), renal biopsy, COVID-19 vaccine, de novo, relapse

## Abstract

**Background:** There is still no consensus about the coronavirus disease 2019 (COVID-19) vaccine-associated glomerular disease (CVAGD). Given the large number of vaccinations administered and the variations in glomerulopathy observed across different countries and regional environments, CVAGD remains an important area of concern. **Aim of study:** We aimed to elucidate the findings of CVAGD within a Taiwanese cohort using biopsy data. Additionally, we endeavored to clarify the presentation of CVAGD. **Methods:** We collected data from patients who underwent renal biopsy from June 2021 to October 2022 at Taichung Veterans General Hospital. Two independent nephrologists meticulously reviewed the charts to exclude cases unrelated to vaccination. **Results:** Initially, a total of 286 patients underwent renal biopsy at our institute. Ultimately, we identified 14 patients with highly suspected CVAGD. All 14 patients exhibited proteinuria and hematuria. The urinary protein-to-creatinine ratio was elevated (median of 2012.1 mg/g; interquartile range (IQR) 25%–IQR 75%: 941.85–3884.1 mg/g) with a median serum creatinine level of 1.71 mg/dL (0.79–5.35). The majority of CVAGD cases were diagnosed as immunoglobulin A (IgA) nephropathy (n = 5, 35.7%), followed by antineutrophil cytoplasmic antibody (ANCA)-related rapidly progressive glomerulonephritis (RPGN) (n = 4, 28.6%). There were only three cases of minimal change disease each: one case of focal segmental glomerulosclerosis, one of membranous glomerulonephritis, and one of lupus nephritis. The culprit of COVID-19 vaccinations was 35.7% (n = 5) of Oxford-AstraZeneca (ChAdOx1-S), 42.9% (n = 6) of Moderna, and 21.4% (n = 3) of BNT162b2. Most patients experienced improvements in renal function. Only two cases of P-ANCA RPGN and one case of IgA nephropathy did not recover. Eighty percent of IgA nephropathy cases had favorable outcomes, but none of the patients with P-ANCA RPGN achieved full recovery. **Conclusions:** IgA nephropathy and ANCA-related RPGN were the most common CVAGD, and all types of COVID-19 vaccines posed a risk for CVAGD. However, further studies are required to confirm causality.

## 1. Introduction

Over the past four years, during the outbreak of the coronavirus disease 2019 (COVID-19) pandemic caused by the severe acute respiratory syndrome (SARS)-coronavirus-2 (SARS-CoV-2), many patients have suffered from this devastating illness. The majority of COVID-19 vaccinations used were messenger RNA (mRNA) vaccines, such as Moderna mRNA-1273 and BioNTech BNT162b2, as well as viral vector-based COVID-19 vaccines like Oxford-AstraZeneca (ChAdOx1-S). Following the introduction of COVID-19 vaccinations, both the severity and mortality rates have declined. However, in light of the large number of vaccinations administered (more than 12.7 billion doses across 184 countries, according to data collected by Bloomberg Vaccine Tracker on 5 October 2022), emerging evidence indicates a potential association between post-COVID-19 vaccination and kidney injury, encompassing acute kidney injury, chronic kidney disease, and glomerular diseases (GDs), which is identified as a COVID-19 vaccine-associated glomerular disease (CVAGD).

Regarding CVAGD, there is still no consensus on its causal effect. Two large observational studies [1,2] have suggested that COVID-19 vaccination is not associated with a higher risk of de novo [1,2] or recurrent from pre-existing [2] glomerular disease. However, with the increased administration of COVID-19 mRNA vaccines (Moderna mRNA-1273 and BioNTech BNT162b2), more and more emerging cases of de novo and relapse of pre-existing glomerular disease have been reported [3,4,5,6,7,8,9,10,11,12,13,14,15]. Therefore, the close monitoring and reporting of CVAGD cases are essential.

Furthermore, even though immunoglobulin A (IgA) nephropathy is the most common glomerular disease in the world, it still exhibits significant heterogeneity in epidemiology, clinical presentation, renal progression, and long-term outcomes in different ethnic populations [16,17]. In the Taiwanese population, only case report for adults [18] and one case series for children [19] were reported regarding CVAGD. There has been no population-based analysis for adults in Taiwan regarding CVAGD. Additionally, many Taiwanese individuals have received viral vector-based COVID-19 vaccines, such as the ChAdOx1-S vaccine. Reports on CVAGD related to the ChAdOx1-S COVID-19 vaccine are much less frequently reported, possibly due to selection bias. Therefore, we aimed to present the findings of CVAGD in this Taiwanese cohort.

## 2. Materials and Methods

### 2.1. Definition of Population

We collected data of patients who underwent renal biopsy at our institute, Taichung Veterans General Hospital. The yearly number of renal biopsies performed in our institute ranged from 250 to 300. Over the past 30 years, we have accumulated a total of more than 9000 renal biopsy, making us the leading institution in Taiwan in terms of biopsies.

During the COVID-19 outbreak, we specifically collected data on renal biopsy cases. In Taiwan, the first COVID-19 vaccine was administered to the general population in June 2021. We conducted a chart review from 1 November 2022 to 30 November 2022 of renal biopsies performed from 1 June 2021 to 31 October 2022. The renal biopsies were performed from 1 June 2021 to 31 October 2022. All information could not identify individual participants during and after data collection. All participants included in the study had no previous history of primary or secondary glomerular disease prior to the renal biopsy. Two independent nephrologists meticulously reviewed the charts to exclude cases unrelated to vaccination (e.g., those caused by painkillers or underlying lupus nephritis). Renal transplant recipients were also excluded from the study. Finally, cases of CVAGD were collected for further analysis. CVAGD was defined as renal biopsy-proven GD occurring after the onset of symptoms and signs. This study was conducted at Taichung Veterans General Hospital and was approved by the institutional review committee (Approval numbers: CE22319A and CE21377A, TCVGH. Approved on 6 October 2021). Since the data were anonymized, informed consent was not required for this study.

### 2.2. Types of COVID-19 Vaccine in Taiwanese Cohort

Data from the Taiwan Centers for Disease Control (Supplementary Table S1) revealed that 49.2% of the population received ChAdOx1-S vaccinations (deployed since 22 March 2021) (viral vector), 24.4% received Moderna mRNA-1273 (deployed since 9 June 2021) (nucleic acid vaccine), and 20.9% of the population received BioNTech BNT162b2 vaccines (deployed since 22 September 2021) (nucleic acid vaccine).

### 2.3. Definition of Proteinuria, Hematuria and Treatment Response

We defined proteinuria as the presence of protein in urine detected by any of the following criteria: daily urine protein levels exceeding 150 mg/day, or a UPCR greater than 150 mg/g. Similarly, the definition of hematuria relied on positive findings in urine sediment microscopy (defined as the presence of three or more RBCs per high-power field in a spun urine sediment), without confirmed causes such as urinary tract infection or malignancy. The complete response to treatment was characterized by improvements in both serum creatinine levels (SCr) and proteinuria, the same as or better than the latest data recorded before the administration of the vaccine. Cases where only partial improvement is observed (i.e., the values are not the same as or better than the latest data) are defined as a partial response.

### 2.4. Data Collection

For those included in this target population, we collected baseline data, including age (in years), gender, and underlying diseases (hypertension, diabetes mellitus, hyperlipidemia, chronic kidney disease, coronary arterial disease, hepatitis B, and hepatitis C). Blood laboratory data were also collected, including white blood cell count (/cumm), hemoglobin level (g/dL), platelet count (10^3^/cumm), kappa/lambda ratio, hepatitis markers, aspartate aminotransferase level (U/L), alanine aminotransferase level (U/L), blood urea nitrogen level (mg/dL), SCr (mg/dL), serum albumin level (g/dL), total protein level (g/dL), lactate dehydrogenase level (mg/dL), low-density lipoprotein cholesterol level (mg/dL), triglyceride level (mg/dL), uric acid level (mg/dL), fasting glucose level (mg/dL), and hemoglobin A1c level (%). In the protocol of renal biopsy at our institution, we also collect 24 h creatinine clearance (ml/min) for every patient. Urine samples were checked for spot urine protein-to-creatinine ratio (mg/g) (UPCR), spot urine albumin-to-creatinine ratio (mg/g) (UACR), proteinuria, and hematuria. Immunological data were also collected for analysis, including immunoglobulins (IgG, IgA, IgM, and IgE), anti-nuclear antibody (ANA), anti-glomerular basement membrane antibody (anti-GBM), and antineutrophil cytoplasmic antibody (ANCA) (including myeloperoxidase (MPO) and proteinase 3 (PR3)). Regarding COVID-19 vaccination, we collected information on all brands of COVID-19 vaccines received prior to renal biopsy, including mRNA vaccines (Moderna mRNA-1273 and BioNTech BNT162b2) (BioNTech) and viral vector-based COVID-19 vaccines (Oxford-AstraZeneca (ChAdOx1-S)). We recorded any symptoms or signs before renal biopsy, the duration between vaccination and the onset of symptoms (in days), the duration between the first vaccination and renal biopsy (in days), and the number of vaccinations received per person. Pathological diagnoses were also collected for analysis.

We also collected treatment data for the patients, including medications, blood purification techniques (plasmapheresis and hemodialysis), and immunosuppressants. We refrained from conducting follow-up renal biopsies to assess the outcomes of CVAGD due to the invasive nature of the procedure. Nevertheless, we maintained regular renal function follow-ups, which included monitoring UPCR and SCr levels.

### 2.5. Statistical Analyses

Due to the limited number of cases, we present the median along with the interquartile range (IQR) for continuous variables. To account for any skewed distribution, we also provided the data range (minimum and maximum). All statistical analyses were performed using the SPSS statistical software package, version 17.0 (Chicago, IL, USA). A *p*-value of less than 0.05 was considered statistically significant.

## 3. Results

### 3.1. Patient Selection Algorithms

**Figure 1:**
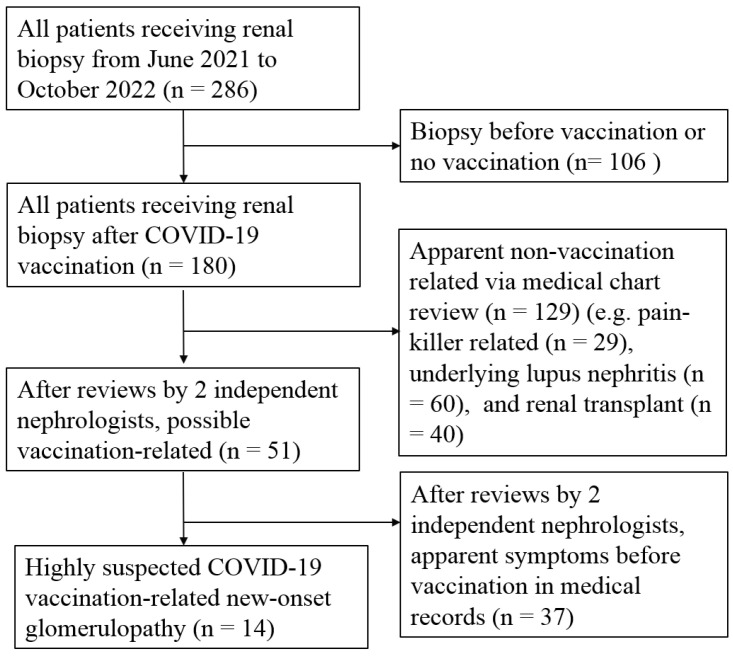
Algorithm of patients’ selection with COVID-19 vaccination-related new-onset glomerulopathy.

During the study period, which spanned from June 2021 to October 2022, a total of 286 patients underwent renal biopsy at our institute. We excluded 106 patients who had undergone renal biopsy prior to receiving the COVID-19 vaccine or did not receive the vaccine. Additionally, 129 patients were excluded based on apparent non-vaccine-related indications for renal biopsy as documented in the medical record, including pain-killer-related (n = 29), underlying lupus nephritis (n = 60), and renal transplant (n = 40). Two independent nephrologists conducted a thorough medical review to identify patients with apparent indications for renal biopsy related to COVID-19, such as symptoms or signs occurring after receiving the vaccine. Ultimately, we identified 14 patients who exhibited GD following COVID-19 vaccine administration (highly suspected CVAGD) and included them in further analysis. A detailed algorithm outlining the selection process is summarized in Figure 1.

### 3.2. Baseline Characteristics of Patients Who Exhibited Highly Suspected CVAGD

In Table 1, among the 14 patients diagnosed with CVAGD, they exhibited a relatively young age profile (50 years old of median age, IQR 25–75%: 33.75–64.75), and only a minority had pre-existing chronic conditions before renal biopsy. Specifically, 14.3% had diabetes mellitus, 14.3% had hypertension, 14.3% had hyperlipidemia, 21.4% had chronic kidney disease, and only one patient (7.1%) had coronary artery disease. Metabolic data did not reveal any significant abnormalities, including fasting glucose (mg/dL) (89.0, 76.0–96.5), glycated hemoglobin (%) (5.4, 5.18–5.85), low-density lipoprotein cholesterol (mg/dL) (104.0, 96.5–144.0), triglycerides (mg/dL) (89.5, 76.0–96.5), and uric acid (mg/dL) (7.35, 5.25–9.18). The complete blood count data showed relatively normal values: white blood cell count (/cumm) (7170, 5327–11,275), hemoglobin (g/dL) (11.9, 8.75–12.33), and platelet count (×10^3^/cumm) (218.5, 181.75–265.25). Liver function tests did not indicate any apparent abnormalities, with aspartate aminotransferase levels (U/L) (21.0, 15.5–35.5) and alanine aminotransferase levels (U/L) (20.5, 11.5–27.3). More than 20% of patients were diagnosed with hepatitis B (21.4%) and hepatitis C (28.6%).

All 14 patients exhibited proteinuria and hematuria. The UPCR was elevated, reaching 2012.1 (941.85–3884.1) mg/g, and the UACR was up to 1789.2 (894.35–3158.33) mg/g. Renal function was impaired, with a serum creatinine level of 1.71 (0.79–5.35) mg/dL and a 24 h creatinine clearance of 79.3 (11.13–103.16) mL/min. Anti-nuclear antibody was positive in 35.7% of patients, and ANCA was positive in 28.6% of patients. Among the renal biopsy findings, the majority of CVAGD cases were diagnosed as IgA nephropathy (n = 5, 35.7%), followed by ANCA-related rapidly progressive glomerulonephritis (RPGN) (n = 4, 28.6%). Other CVAGDs were less common, with minimal change disease (MCD), focal segmental glomerulosclerosis (FSGS), membranous glomerulonephritis (MN), and lupus nephritis.

### 3.3. Detailed Information of COVID-19 Vaccines

All three common COVID-19 vaccines were administered in this population, as indicated in Table 2. Among the 14 patients, a total of 28 doses of COVID-19 vaccines were administered. Regarding the culprit of COVID-19 vaccination (the closest vaccine to the biopsy date) (total n = 14), 35.7% (n = 5) received ChAdOx1-S, 42.9% (n = 6) received Moderna mRNA-1273, and only 21.4% (n = 3) received BioNTech BNT162b2. The mean duration from vaccination to the onset of symptoms was 55 days (IQR = 2–90). However, some patients reported experiencing hematuria or foamy urine just one day after vaccination. On average, each of the 14 patients received 2.0 (IQR = 1.25–4.50) vaccinations. The specific COVID-19 vaccines received by each patient prior to renal biopsy are presented in Table 2.

Only four patients (cases 1, 5, 8, and 14) with CVAGD received the first dose of COVID-19 vaccines. Out of these four patients, three received the ChAdOx1-S COVID-19 vaccine once, and one received the Moderna mRNA-1273 vaccine once. The majority of patients (n = 10, 71.4%) with CVAGD have received at least two doses of COVID-19 vaccines.

### 3.4. Treatment

Before presenting the treatment outcomes, we need to clarify that not all patients are undergoing typical treatments.

For case 2, initial treatment consisted of prednisolone 40 mg per day. However, due to a poor response, the clinician augmented the regimen with cyclosporine. This treatment approach aligns with recommendations that advocate for glucocorticoid monotherapy, which achieves complete remission in 80 to over 95 percent of adults with MCD. For patients unable to tolerate, contraindicated for, or unwilling to take high-dose glucocorticoids, glucocorticoid-sparing regimens such as calcineurin inhibitors (CNIs; cyclosporine or tacrolimus) or mycophenolate mofetil/enteric-coated mycophenolate sodium (MMF/EC-MPS) alongside reduced-dose glucocorticoids are viable alternatives. We adjusted the treatment regimen in Table 3 to include an angiotensin receptor blocker (ARB) and prednisolone 40 mg initially, followed by cyclosporine 100 mg.

For case 4, pathology revealed 50% interstitial fibrosis and tubular atrophy (IFTA), and the patient was already undergoing hemodialysis. At 68 years old, the patient opted against pulse therapy and was only prescribed low-dose immunosuppressants, including low-dose prednisolone and cyclophosphamide.

For case 5, pathology indicated M0E0S0T0-C0 of IgA nephropathy, with mild arteriosclerosis. Consequently, the clinician chose low-dose steroid therapy only (due to M0E0S0T0-C0) while concurrently managing blood pressure.

For case 10, despite having FSGS, the patient presented with a serum creatinine level of 4.56 mg/dL. Pathology revealed FSGS with six global glomerulosclerosis and one segmental glomerulosclerosis in all eight glomeruli. Hence, the clinician opted against steroid treatment due to the significant chronicity of the condition.

### 3.5. Treatment Outcome of CVAGD

Treatment and follow-up were conducted for patients who reported glomerulopathy related to post-COVID-19 vaccination (Table 3). Most patients experienced improvements in renal function, including reductions in SCr and UPCR, after receiving timely treatment. Cases of complete improvement were observed in patients with minimal change disease (case 2), lupus nephritis (case 3), IgA nephropathy (cases 6, 7, and 8), and membranous glomerulonephritis (case 11). Cases of partial improvement were observed in patients with P-ANCA RPGN (cases 9 and 12), focal segmental glomerulosclerosis (case 10), and IgA nephropathy (case 13). However, some patients did not show any recovery, including those with P-ANCA RPGN (cases 4 and 14) and IgA nephropathy (case 5).

In terms of pathological diagnosis, patients with IgA nephropathy generally had favorable outcomes. Among the five patients with IgA nephropathy (cases 5, 6, 7, 8, and 13), only one (case 5) did not experience any improvement, while three patients (cases 6, 7, and 8) achieved full recovery of renal function. On the other hand, patients with P-ANCA RPGN (cases 4, 9, 12, and 14) had a poor prognosis. None of the patients with P-ANCA RPGN achieved full recovery, with half of them (cases 9 and 12) experiencing partial improvement and the other half (cases 4 and 14) showing no improvement at all.

## 4. Discussion

Our report represents the first study to illustrate GD following COVID-19 vaccine administration (highly suspected CVAGD) in the adult population of Taiwan. Our data are in line with a previous population-based analysis conducted in Canada [20]. In that study, a centralized clinical and pathology registry spanning from 2000 to 2020 identified 1105 adult patients in British Columbia, Canada, with biopsy-proven glomerular disease. The absolute increase in the 30-day relapse prevalence of CVAGD ranged from 1% to 2% in ANCA-related glomerulonephritis, MCD, MN, or FSGS and from 3% to 5% in IgA nephropathy or lupus nephritis. In our dataset, we observed a similar pattern of CVAGD, which encompassed a de novo of IgA nephropathy (n = 5), ANCA-related RPGN (n = 4), MCD (n = 2), MN (n = 1), FSGS (n = 1), and lupus nephritis (n = 1).

The impact of the COVID-19 vaccine dose on CVAGD warrants further investigation. There is a possibility that the second dose of the vaccine could have an adverse effect on glomerular disease due to a heightened immune response from the booster dose. In a retrospective population-level cohort study [20], the first dose of the COVID-19 vaccine was not associated with an increased relapse chance (HR = 0.67; 95% CI, 0.33 to 1.36). However, exposure to a second or third dose was linked to a two-fold risk of relapse (HR = 2.23; 95% CI, 1.06 to 4.71). In our study, 28.6% of CVAGD cases occurred after the first vaccine dose, while the majority (71.4%) occurred after receiving more than one dose. Our findings are consistent with another study conducted in the USA, where 23% of cases were reported after the first dose of vaccination, and 77% occurred after the second dose [21]. This suggests that receiving multiple doses of the COVID-19 vaccine may be associated with a higher prevalence of CVAGD. Nevertheless, attention should also be given to the chance of CVAGD after the first dose of vaccination, as observed in cases of IgA nephropathy (2 cases) and P-ANCA-related RPGN (1 case) in our study. The probability associated with the first dose of the COVID-19 vaccine cannot be overlooked, which aligns with the findings of an editorial published in 2021 [22]. In that publication, a summarized table showed that 50% of cases (n = 13) were reported after the first dose of COVID-19 vaccination, and the remaining 50% (n = 13) occurred after the second dose. Furthermore, a questionnaire survey conducted in Japan also demonstrated that 85.2% of cases (n = 23) observed nephrotic syndrome onset following vaccination with BNT162b2 (BioNTech), with incidences reported after both the first (n = 8) and second (n = 15) doses of the vaccine [21]. In summary, our study highlights the importance of considering the probability of CVAGD even after the first dose of the COVID-19 vaccine. Additional research is needed to understand the impact of multiple vaccine doses on CVAGD to ensure the safe administration of vaccinations.

The impact of different types of COVID-19 vaccination on CVAGD risk requires further investigation. In our data, among the culprit COVID-19 vaccinations (total n = 14), 35.7% (n = 5) received ChAdOx1-S, 42.9% (n = 6) received Moderna mRNA-1273, and only 21.4% (n = 3) received BioNTech BNT162b2. However, it is important to note that much fewer reported cases (2 of 26, 7.7%) of CVAGD were associated with the ChAdOx1-S vaccine in one case series [22]. Similarly, in another case series study (24), the Pfizer-BioNTech BNT162b2 vaccine was the most commonly administered vaccine (15 of 27 patients, 55%) for patients with CVAGD, followed by Moderna mRNA-1273 (8 of 27 patients, 30%), with only 3 patients (11%) receiving the ChAdOx1-S vaccine. Contrary to the findings of these studies, our data still showed that the ChAdOx1-S vaccine did not have the lowest risk for CVAGD. We believe that the discrepancies between our data and those from other studies may be attributed to selection bias. It is worth considering that during the first year of the COVID-19 outbreak in Taiwan, the majority of vaccines administered were ChAdOx1-S. As a result, at the time of our recruitment, we had a higher number of renal biopsy cases from individuals who received the ChAdOx1-S vaccinations. Data from the Taiwan Centers for Disease Control (Supplementary Table S1) revealed that 49.2% of the population received ChAdOx1-S vaccinations (deployed since 22 March 2021), 24.4% received Moderna mRNA-1273 (deployed since 9 June 2021), and 20.9% of the population received BioNTech BNT162b2 vaccines (deployed since 22 September 2021). Consequently, in our cohort, with a higher proportion of ChAdOx1-S vaccinations, it was observed that ChAdOx1-S vaccinations did not show a lower risk for CVAGD compared to Moderna mRNA-1273 and BioNTech BNT162b2 vaccines. This suggests that the risk of CVAGD may not solely be attributed to the type of COVID-19 vaccine administered, and other factors should be considered in the evaluation of CVAGD risk. Further research is needed to clarify the association between different COVID-19 vaccines and CVAGD incidence.

The most common type of CVAGD in our cohort is IgA nephropathy, which is consistent with previous reports, including de novo [3,4,23] or relapsing pre-existing [9,10,11] glomerular disease. Relapsing IgA nephropathy following influenza vaccination has also been reported in renal transplant recipients [24,25]. However, the underlying mechanism remains incompletely understood. The most widely accepted explanation involves the production of antiglycan antibodies that cross-react with pre-existing under-galactosylated IgA1 [26]. Additionally, previous studies have indicated that mRNA COVID-19 vaccines may lead to an elevation of circulating galactose-deficient IgA1 (Gd-IgA1) [27]. Subsequent to COVID-19 vaccination, antibody titers in patients increase exponentially, possibly triggering outbreaks of IgA nephropathy. Previous analyses indicated that the majority of IgA nephropathy cases occurred after receiving the second vaccine dose [22], which is in line with our own results, where 40% of cases were related to the first dose, and 60% were associated with receiving more than one vaccine dose. For patients with CVAGD-IgA nephropathy who only received the first dose, we cannot exclude the possibility that they already had a relatively active status of IgA nephropathy but without formal confirmation.

The underlying mechanism of ChAdOx1-S vaccine-related IgA nephropathy remains unknown, and the number of reported cases is further limited. According to a previous report [28], most IgA nephropathy cases after COVID-19 vaccines are associated with mRNA vaccines, with only 6.3% linked to the ChAdOx1-S vaccine. Similarly, in another case report and literature review published last month [29], only 7.7% (4 of 52) of IgA nephropathy cases were related to the ChAdOx1-S vaccine. In our dataset, most IgA nephropathy cases were related to mRNA vaccines (four of five, 80%), with only one patient (20%) associated with the ChAdOx1-S vaccine. This suggests that there may be differences in the risk of CVAGD-IgA nephropathy based on the type of COVID-19 vaccine administered, and further research is needed to elucidate the underlying mechanisms and better understand these associations.

The second-most-common CVAGD in our cohort is ANCA-related RPGN. COVID-19 vaccine-related CVAGD has also been reported in case reports [30,31,32]. According to a review article on PUBMED [30], covering the period from 1 January 2020 to 20 April 2022, there have been 22 articles, including 27 cases that fulfill the criteria for de novo ANCA-related RPGN reported in temporal association with COVID-19 vaccination. The exact mechanism of ANCA-related RPGN due to COVID-19 vaccine remains unknown, but it is hypothesized that an enhanced immune response after booster vaccination and the presence of HLA-DR+ monocytes could trigger the production of observed MPO- and PR3-ANCA autoantibodies [33,34,35]. The prognosis may be good with prompt treatment [32]. However, in our cohort, we only observed partial improvement due to delayed diagnosis.

There are some limitations in this study. Firstly, the main limitation is the weak correlation between vaccine administration and the development of glomerulopathy. However, there was a noticeable temporal link between the symptoms and signs of glomerulopathy and the administration of vaccinations. The timing of symptom onset shortly after vaccination should be considered as the inciting event (i.e., a disease that occurs ≤30 days after an administered vaccine). Additionally, we conducted a comprehensive investigation to rule out secondary causes of glomerulopathy, such as medications, infections, and autoimmune diseases. All studies regarding COVID-19 vaccine-related complications have this similar limitation. In this study, all possible cases were reviewed from two independent nephrologists; they ensured that any new symptoms or signs were absent before the vaccine, verified through medical records and laboratory data. Secondly, the number of cases in this study is still relatively small. However, despite these limitations, we believe that our data still hold value in advocating for renal function checks before and after COVID-19 vaccine administration. Finally, CVAGD is often underdiagnosed because there is no guideline recommending routine renal function follow-up after COVID-19 vaccine administration. We can only rely on patients’ symptoms and signs (leg edema, hematuria, or foamy urine) and incidental findings of renal dysfunction. Not every glomerular disease leads to symptoms. Based on these conditions, we can perform a renal biopsy. Therefore, we believe CVAGD is underdiagnosed.

## 5. Conclusions

The most commonly reported cases of CVAGD were associated with IgA nephropathy and ANCA-related RPGN. It is essential to recognize that all types of vaccines pose a risk for CVAGD. However, when RPGN is not present, the outcomes are generally favorable with timely diagnosis and treatment.

## Figures and Tables

**Table 1:** Baseline characteristics of patients with new-onset glomerulonephropathy.

Characteristics	All Cases (n = 14) (Median, IQR 25–75%)
Age (years old)	50, 33.75–64.75
Male gender (n, %)	6 (42.9%)
Underlying disease (n, %)	
Diabetes mellitus	2 (14.3%)
Hypertension	2 (14.3%)
Hyperlipidemia	2 (14.3%)
Chronic kidney disease	3 (21.4%)
Coronary artery disease	1 (7.1%)
Which COVID-19 vaccination-related	
Oxford-AstraZeneca (ChAdOx1-S)	5 (35.7%)
Moderna (mRNA-1273)	6 (42.9%)
Pfizer-BioNTech (BNT162b2)	3 (21.4%)
Duration between vaccination and symptoms (days)	55, 2–90
Duration between the first dose vaccination and renal biopsy (days)	5.5, 2.0–20
Numbers of vaccination per person	2, 1.25–4.50
White blood cell (/cumm)	7170, 5327–11,275
Hemoglobin (g/dL)	11.9, 8.75–12.33
Platelet count (×10^3^/cumm)	218.5, 181.75–265.25
Serum immunoglobulin (Ig)	
IgG	1208, 1075.8–1515.0
IgA	324.2, 284.1–487.5
IgM	83.5, 63.7–113.6
IgE	83.1, 41.4–402.0
Kappa/lambda ratio	1.58, 0.44–2.02
Hepatitis B	3 (21.4%)
Hepatitis C	4 (28.6%)
Aspartate aminotransferase (U/L)	21.0, 15.5–35.5
Alanine aminotransferase (U/L)	20.5, 11.5–27.3
Blood urea nitrogen (mg/dL)	28.0, 15.8–59.0
Serum creatinine (mg/dL)	1.71, 0.79–5.35
24 h Creatinine clearance (ml/min)	79.3, 11.13–103.16
Spot urine protein/creatinine ratio (mg/g)	2012.1, 941.85–3884.1
Spot urine albumin/creatinine ratio (mg/g)	1789.2, 894.35–3158.33
Proteinuria	0 (2, 14.3%), 1+ (3, 21.4%), 2+ (5, 35.7%), 3+ (4, 28.6%)
Hematuria	1+ (3, 21.4%), 2+ (2, 14.3%), 3+ (9, 64.3%)
Serum albumin (g/dL)	3.9, 3.33–4.10
Total protein (g/dL)	6.85, 6.10–7.20
Lactate dehydrogenase (mg/dL)	174.5, 160.0–243.3
Low-density lipoprotein cholesterol (mg/dL)	104.0, 76.5–144.0
Triglyceride (mg/dL)	89.5, 52.75–135.50
Uric acid (mg/dL)	7.35, 5.25–9.18
Fasting glucose (mg/dL)	89.0, 76.0–96.5
Hemoglobin A1c (%)	5.4, 5.18–5.85
Anti-nuclear antibody (positive)	5 (35.7%)
Anti-glomerular basement membrane antibody (positive)	0
Antineutrophil cytoplasmic antibody (positive)	4 (28.6%)
Myeloperoxidase	4 (28.6%)
Serine proteinase 3	0
Pathological diagnosis	
IgA nephropathy	5 (35.7%)
ANCA-related rapid progressive glomerulonephritis	4 (28.6%)
Minimal change disease	3 (21.6%)
Focal segmental glomerulosclerosis	1 (7.1%)
Membranous glomerulonephritis	1 (7.1%)
Lupus nephritis	1 (7.1%)

**Table 2:** Detailed vaccine-related information.

**Case**	**Age**	**Gender**	**Vaccine ^a^**	**The Closest Vaccination to the Initial Presentation**	**1st Vaccination to the Initial Presentation**	**Pathological Report**
1	49	F	A	3	3	MCD ^i^
2	59	M	AA	20	110	MCD ^i^
5	51	F	A	20	20	IgA nephropathy (M0E0S0T0-C0), mild arteriosclerosis and focal chronic IFTA ^d^ (10%)
6	27	F	AM	2	92	IgA nephropathy (M0E0S0T0-C0) and mild glomerulomegaly
7	24	F	BB	1	91	IgA nephropathy (Haas’s subclass 1; Oxford classification: M0E0S0T0)
8	47	F	M	14	14	IgA nephropathy (M0E1S0T0)
13	67	M	MMMM	90	250	IgA nephropathy (M0E1S0T2-C0)
4	68	F	AA	120	210	P-ANCA ^c^ (Pauci-immune necrotizing crescentic GN with 57% cellular/fibrocellular crescent; IFTA ^d^ 50%)
9	60	M	BB	2	92	P-ANCA ^c^; (cellular/fibrocellular crescent: 10/24 glomeruli; fibrous crescent: 4/24 glomeruli; globally obsolete: 11/24)
12	64	M	MMM	7	187	P-ANCA ^c^ (38% cellular/fibrocellular crescent), gg 3/21
14	74	F	A	115	115	P-ANCA ^c^ (gg 6/12, sg 6/12)
3	19	M	MM	90	180	Diffuse lupus glomerulonephritis, ISN/RPS ^b^ class IV (modified NIH score AI 7/24; CI 0/12)
10	36	M	BB	14	104	FSGS ^e^, gg ^f^ 1/8, sg ^g^ 1/8
11	37	F	AAM	2	182	MN ^h^

^a^ A: Oxford-AstraZeneca (ChAdOx1-S)-a; M: Moderna mRNA-1273; B: Pfizer-BioNTech BNT162b2) (Pfizer-BioNTech); ^b^ ISN/RPS: International Society of Nephrology and Renal Pathology Society; ^c^ P-ANCA: perinuclear antineutrophil cytoplasmic antibody; ^d^ IFTA: interstitial fibrosis and tubular atrophy; ^e^ FSGS: focal segmental glomerulosclerosis; ^f^ gg: global glomerulosclerosis; ^g^ sg: segmental glomerulosclerosis; ^h^ MN: membranous glomerulonephritis; ^i^ MCD: minimal change disease.

**Table 3:** Treatment and follow-up patients who reported post-COVID-19 vaccination-related glomerulopathy.

Case	Age	Diagnsosi	SCr ^d^	UPCR ^e^	Treatment	Outcome	Time to Response	Follow-Up
1	49	MCD	1.2	4779.56	Observation	Loss of follow-up	Loss of follow-up	Loss of follow-up
2	59	MCD	0.96	3882.26	ARB ^g^ and PD ^c^ 40 mg initially, followed by CsA 100 mg	Full improvement: SCr ^d^ = 1.0 mg/dL, serum albumin (2.4→4.4 g/dL), UPCR ^e^ (10,523→91.46 mg/g)	2 weeks	6 months
3	19	Diffuse lupus glomerulonephritis	2.09	1728.16	PD ^c^ 8 mg QD, Myfrotic 4# bid	Full improvement: UPCR ^e^ (1728→115 mg/g); SCr ^d^ = 2.09→1.19 mg/dL	1 month	6 months
4	68	P-ANCA ^a^	hemodialysis	5941.51	PD ^c^ 8 mg QD, Cyclophosphamide 50 mg qw	No improvement: still undergoing HD	No response	6 months
5	51	IgA nephropathy	1.8	1299.53	PD ^c^ 5 mg qd, SGLT2i ^h^, and ARB ^g^	No improvement: UPCR ^e^ (1299→1649 mg/g); SCr ^d^ = (1.64→1.78 mg/dL)	No response	9 months
6	27	IgA nephropathy	0.7	273.65	Observation	Full improvement: SCr ^d^ (0.53→0.77 mg/dL), UPCR ^e^ (337→101 mg/g)	1 month	6 months
7	24	IgA nephropathy	0.75	584.16	Observation	Full improvement: SCr (0.65→0.72 mg/dL), UPCR ^e^ (584→80 mg/g)	3 weeks	7 months
8	47	IgA nephropathy	0.81	238.62	ARB ^g^	Full improvement: SCr ^d^ (0.70→0.89 mg/dL), UPCR ^e^ (1314→556 mg/g)	1 month	8 months
9	60	P-ANCA ^a^	5.17	3885.77	Plasmapheresis × 5, methylprednisolone pulse therapy 500 mg × 3 days then PD ^c^ 40 mg qd, temporary hemodialysis five times	Partial improvement: MPO ^f^ (134→18); SCr ^d^ (9.03→6.0 mg/L), UPCR ^e^ (3885→3356 mg/g)	2 months	5 months
10	36	FSGS ^b^	4.56	2012.12	ARB ^g^	Partial improvement: SCr ^d^ (6.28→4.79→3.57 mg/dL); UPCR (2012→1007 mg/g)	2 weeks	9 months
11	37	Membranous glomerulonephritis	0.74	3314.69	PD ^c^ 50 mg qd, Cyclophosphamide 50 mg TID	Full improvement: SCr ^d^ (0.71→0.71 mg/dL), UPCR ^e^ (2345→ 200 mg/g)	3 months	9 months
12	64	P-ANCA ^a^	2.59	1976.73	Plasmapheresis × 10, PD 20 mg qd	Partial improvement: SCr ^d^ (1.18→2.59→3.93→2.78 mg/dL); UPCR ^e^ (1976–4271 g/mg), MPO ^f^ (134→21)	2 months	6 months
13	67	IgA nephropathy	1.62	469.5	ARB ^g^	Partial improvement: SCr ^d^ (2.87→1.43 mg/dL); UPCR ^e^ (1049→120 mg/g)	1 month	6 months
14	74	P-ANCA ^a^	6.33	2247.02	Plasmapheresis × 5, methylprednisolone pulse therapy 500 mg × 3 days then PD ^c^ 10 mg qd,	No improvement: SCr ^d^ (6.33→6.14 mg/dL), UPCR ^e^ (2247→2441 mg/g), MPO ^f^ (13→3.2)	No response	6 months

^a^ P-ANCA: Perinuclear antineutrophil cytoplasmic antibody; ^b^ FSGS: focal segmental glomerulosclerosis; ^c^ PD: prednisolone; ^d^ SCr: serum creatinine; ^e^ UPCR: Urine protein/creatinine ratio; ^f^ MPO: myeloperoxidase; ^g^ ARB: angiotensin II receptor blocker; ^h^ SGLT2i: sodium–glucose cotransporter 2 inhibitors.

## Data Availability

The original contributions presented in the study are included in the article/Supplementary Materials; further inquiries can be directed to the corresponding authors.

## Supplementary Materials

The following supporting information can be downloaded at: https://www.mdpi.com/article/10.3390/jcm13154494/s1 Table S1: All COVID-19 vaccinations until 2023/11/01.

## References

[1] Diebold M., Locher E., Boide P., Enzler-Tschudy A., Faivre A., Fischer I., Helmchen B., Hopfer H., Kim M.J., Moll S. (2022). Incidence of new onset glomerulonephritis after SARS-CoV-2 mRNA vaccination is not increased. Kidney Int..

[2] Cheng F.W.T., Wong C.K.H., Qin S.X., Chui C.S.L., Lai F.T.T., Li X., Wan E.Y.F., Chan E.W., Au C.H., Ye X. (2023). Risk of glomerular diseases, proteinuria and hematuria following mRNA (BNT162b2) and inactivated (CoronaVac) SARS-CoV-2 vaccines. Nephrol. Dial. Transplant. Off. Publ. Eur. Dial. Transpl. Assoc.—Eur. Ren. Assoc..

[3] Anderegg M.A., Liu M., Saganas C., Montani M., Vogt B., Huynh-Do U., Fuster D.G. (2021). De novo vasculitis after mRNA-1273 (Moderna) vaccination. Kidney Int..

[4] Tan H.Z., Tan R.Y., Choo J.C.J., Lim C.C., Tan C.S., Loh A.H.L., Tien C.S., Tan P.H., Woo K.T. (2021). Is COVID-19 vaccination unmasking glomerulonephritis?. Kidney Int..

[5] Lebedev L., Sapojnikov M., Wechsler A., Varadi-Levi R., Zamir D., Tobar A., Levin-Iaina N., Fytlovich S., Yagil Y. (2021). Minimal Change Disease Following the Pfizer-BioNTech COVID-19 Vaccine. Am. J. Kidney Dis. Off. J. Natl. Kidney Found..

[6] Maas R.J., Gianotten S., van der Meijden W.A.G. (2021). An Additional Case of Minimal Change Disease Following the Pfizer-BioNTech COVID-19 Vaccine. Am. J. Kidney Dis. Off. J. Natl. Kidney Found..

[7] D’Agati V.D., Kudose S., Bomback A.S., Adamidis A., Tartini A. (2021). Minimal change disease and acute kidney injury following the Pfizer-BioNTech COVID-19 vaccine. Kidney Int..

[8] Holzworth A., Couchot P., Cruz-Knight W., Brucculeri M. (2021). Minimal change disease following the Moderna mRNA-1273 SARS-CoV-2 vaccine. Kidney Int..

[9] Perrin P., Bassand X., Benotmane I., Bouvier N. (2021). Gross hematuria following SARS-CoV-2 vaccination in patients with IgA nephropathy. Kidney Int..

[10] Negrea L., Rovin B.H. (2021). Gross hematuria following vaccination for severe acute respiratory syndrome coronavirus 2 in 2 patients with IgA nephropathy. Kidney Int..

[11] Rahim S.E.G., Lin J.T., Wang J.C. (2021). A case of gross hematuria and IgA nephropathy flare-up following SARS-CoV-2 vaccination. Kidney Int..

[12] Komaba H., Wada T., Fukagawa M. (2021). Relapse of Minimal Change Disease Following the Pfizer-BioNTech COVID-19 Vaccine. Am. J. Kidney Dis. Off. J. Natl. Kidney Found..

[13] Kervella D., Jacquemont L., Chapelet-Debout A., Deltombe C., Ville S. (2021). Minimal change disease relapse following SARS-CoV-2 mRNA vaccine. Kidney Int..

[14] Schwotzer N., Kissling S., Fakhouri F. (2021). Letter regarding “Minimal change disease relapse following SARS-CoV-2 mRNA vaccine”. Kidney Int..

[15] Aydın M.F., Yıldız A., Oruç A., Sezen M., Dilek K., Güllülü M., Yavuz M., Ersoy A. (2021). Relapse of primary membranous nephropathy after inactivated SARS-CoV-2 virus vaccination. Kidney Int..

[16] Yeo S.C., Goh S.M., Barratt J. (2019). Is immunoglobulin A nephropathy different in different ethnic populations?. Nephrology.

[17] Zhang H., Barratt J. (2021). Is IgA nephropathy the same disease in different parts of the world?. Semin. Immunopathol..

[18] Tseng P.H., Chuang S.H., Pan Y., Shih H.J., Chang C.P., Huang S.H. (2022). Gross hematuria and IgA nephropathy flare-up following the first dose of Moderna vaccine: A case report. Medicine.

[19] Chuang G.T., Lin W.C., Chang L.Y., Tsai I.J., Tsau Y.K. (2023). Pediatric glomerulopathy after COVID-19 vaccination: A case series and review of the literature. J. Formos. Med. Assoc. = Taiwan Yi Zhi.

[20] Canney M., Atiquzzaman M., Cunningham A.M., Zheng Y., Er L., Hawken S., Zhao Y., Barbour S.J. (2022). A Population-Based Analysis of the Risk of Glomerular Disease Relapse after COVID-19 Vaccination. J. Am. Soc. Nephrol. JASN.

[21] Nakagawa N., Maruyama S., Kashihara N., Narita I., Isaka Y. (2022). New-onset and relapse of nephrotic syndrome following COVID-19 vaccination: A questionnaire survey in Japan. Clin. Exp. Nephrol..

[22] Bomback A.S., Kudose S., D’Agati V.D. (2021). De Novo and Relapsing Glomerular Diseases After COVID-19 Vaccination: What Do We Know So Far?. Am. J. Kidney Dis. Off. J. Natl. Kidney Found..

[23] Waldman M., Sinaii N., Lerma E.V., Kurien A.A., Jhaveri K.D., Uppal N.N., Wanchoo R., Avasare R., Zuckerman J.E., Liew A. (2023). COVID-19 Vaccination and New Onset Glomerular Disease: Results from the IRocGN2 International Registry. Kidney360.

[24] McNally A., McGregor D., Searle M., Irvine J., Cross N. (2013). Henoch-Schönlein purpura in a renal transplant recipient with prior IgA nephropathy following influenza vaccination. Clin. Kidney J..

[25] Fischer A.S., Møller B.K., Krag S., Jespersen B. (2015). Influenza virus vaccination and kidney graft rejection: Causality or coincidence. Clin. Kidney J..

[26] Abramson M., Mon-Wei Yu S., Campbell K.N., Chung M., Salem F. (2021). IgA Nephropathy After SARS-CoV-2 Vaccination. Kidney Med..

[27] Nihei Y., Kishi M., Suzuki H., Koizumi A., Yoshida M., Hamaguchi S., Iwasaki M., Fukuda H., Takahara H., Kihara M. (2022). IgA Nephropathy with Gross Hematuria Following COVID-19 mRNA Vaccination. Intern. Med..

[28] Ma Y., Xu G. (2023). New-onset IgA nephropathy following COVID-19 vaccination. QJM Mon. J. Assoc. Physicians.

[29] Mima A., Lee S. (2023). IgA nephropathy after COVID-19 vaccination and analysis of reported cases. Heliyon.

[30] Baier E., Olgemoller U., Biggemann L., Buck C., Tampe B. (2022). Dual-Positive MPO- and PR3-ANCA-Associated Vasculitis Following SARS-CoV-2 mRNA Booster Vaccination: A Case Report and Systematic Review. Vaccines.

[31] Bansal S.B., Rana A.S., Manhas N., Rana A. (2022). Post COVID Vaccination (COVAXIN-BB152 V) Pauci-immune Crescentic Glomerulonephritis. Indian J. Nephrol..

[32] Mai A.S., Tan E.-K. (2022). COVID-19 vaccination precipitating de novo ANCA-associated vasculitis: Clinical implications. Clin. Kidney J..

[33] Iking-Konert C., Vogt S., Radsak M., Wagner C., Hansch G.M., Andrassy K. (2001). Polymorphonuclear neutrophils in Wegener’s granulomatosis acquire characteristics of antigen presenting cells. Kidney Int..

[34] Spies C., Kip M., Lau A., Sander M., Breuer J., Meyerhoefer J., Paschen C., Schumacher G., Volk H., Wernecke K. (2008). Influence of vaccination and surgery on HLA-DR expression in patients with upper aerodigestive tract cancer. J. Int. Med. Res..

[35] Unanue E.R., Beller D.I., Lu C., Allen P. (1984). Antigen presentation: Comments on its regulation and mechanism. J. Immunol..

