# Supplementary material for: De Novo Biopsy-Proven Glomerular Disease Following COVID-19 Vaccination
Source: J Clin Med. 2024 Jul 31;13(15):4494. doi: 10.3390/jcm13154494 (PMC11313347; doi:10.3390/jcm13154494)
Supplement: Supplementary file 1 [file jcm-13-04494-s001.zip › jcm-3105439-supplementary.pdf]

Table S1. All COVID-19 vaccinations until 2023/11/01

**Brand of vaccine**<br>**Status**	**AstraZeneca**		**Moderna**		**Medigen**		**BioNTech**	
Dose	1st	2nd	1st	2nd	1st	2nd	1st	2nd
Medical staffs	594,672	437,933	131,445	194,521	3,166	2,951	11,740	48
Central and Local Government Epidemic Prevention Personnel	221,680	163,829	57,760	46,815	1,019	808	2,712	12
Frontline workers with high exposure risk	116,638	64,090	70,934	62,116	1,183	938	4,910	63
Personnel in institutions and social welfare care system, as well as their caregivers and dialysis patients	339,809	285,734	94,274	62,908	3,514	2,668	9,788	0
Elderly individuals aged 75 and above	560,601	469,079	506,073	302,866	7,430	6,026	16,664	0
Pregnant women	246	131	58,926	43,889	19	7	232	0
Essential personnel to maintain societal operations	466,429	269,059	444,623	233,180	9,302	7,793	23,953	86
Essential personnel to maintain national security and normal operations	132,638	66,951	61,127	34,648	2,756	2,067	12,846	0
Age group 65-74 years old	146,729	155,874	1,486,512	1,459,410	21,954	17,776	44,084	61
Age group 50-64 years old	1,684,185	1,386,398	234,587	92,660	156,786	130,267	877,202	0
Age group 12-17 years old	0	0	0	0	0	0	1,095,777	0
Individuals with special circumstances who need to travel abroad	47,236	31,998	7,140	6,842	210	100	644	0
Others	3,473,519	1,271,685	323,384	125,165	555,310	456,561	3,161,632	0
Subtotal	7,784,382	4,602,761	3,476,785	2,665,020	762,649	627,962	5,262,184	270
